# Advances in mesenchymal stem cells and their derivatives for promoting peripheral nerve regeneration

**DOI:** 10.1093/burnst/tkaf027

**Published:** 2025-05-19

**Authors:** Shuai Wei, Jin Dong, Qian Hu, Jinyu Bai, Xiang Gao, Huajian Shan, Lei Sheng, Jun Dai, Lide Tao, Bing Yan, Xiaozhong Zhou

**Affiliations:** Department of Orthopedics, The Second Affiliated Hospital of Soochow University, No. 1055 Sanxiang Road, Gusu District, Suzhou, Jiangsu 215004, China; Department of Orthopedics, The Second Affiliated Hospital of Soochow University, No. 1055 Sanxiang Road, Gusu District, Suzhou, Jiangsu 215004, China; Health Management Center, The Second Affiliated Hospital of Soochow University, No. 1055 Sanxiang Road, Gusu District, Suzhou, Jiangsu 215004, China; Department of Orthopedics, The Second Affiliated Hospital of Soochow University, No. 1055 Sanxiang Road, Gusu District, Suzhou, Jiangsu 215004, China; Department of Orthopedics, The Second Affiliated Hospital of Soochow University, No. 1055 Sanxiang Road, Gusu District, Suzhou, Jiangsu 215004, China; Department of Orthopedics, The Second Affiliated Hospital of Soochow University, No. 1055 Sanxiang Road, Gusu District, Suzhou, Jiangsu 215004, China; Department of Orthopedics, The Second Affiliated Hospital of Soochow University, No. 1055 Sanxiang Road, Gusu District, Suzhou, Jiangsu 215004, China; Department of Orthopedics, The Second Affiliated Hospital of Soochow University, No. 1055 Sanxiang Road, Gusu District, Suzhou, Jiangsu 215004, China; Department of Orthopedics, The Second Affiliated Hospital of Soochow University, No. 1055 Sanxiang Road, Gusu District, Suzhou, Jiangsu 215004, China; Department of Orthopedics, The Second Affiliated Hospital of Soochow University, No. 1055 Sanxiang Road, Gusu District, Suzhou, Jiangsu 215004, China; Department of Orthopedics, The Second Affiliated Hospital of Soochow University, No. 1055 Sanxiang Road, Gusu District, Suzhou, Jiangsu 215004, China

**Keywords:** Mesenchymal stem cells, Peripheral nerve injury, Exosomes, Mitochondria, Nerve regeneration

## Abstract

Peripheral nerve injury constitutes a complex neurotraumatic pathology characterized by mechanical disruption of neural integrity, manifesting as multimodal sensorimotor deficits and impaired neuromuscular coordination. The primary clinical interventions include surgical tension-free suturing of the severed nerve ends and autologous nerve transplantation. Despite these interventions, patients often experience complications, and the outcomes are not entirely satisfactory for either patients or clinicians. Mesenchymal stem cells (MSCs) have gradually become a novel therapeutic option, with burgeoning preclinical evidence elucidating their multimodal therapeutic potential in peripheral nerve reconstruction. This research has produced promising outcomes, contributing to both fundamental research and translational medicine. However, a comprehensive synthesis of the roles of MSCs and their derivatives in nerve regeneration is still lacking. This article presents a review of the current research advancements in this area, aiming to encourage further investigations and therapeutic applications of MSCs and their derivatives in peripheral nerve injury and regenerative medicine.

Highlights

- Compared with other mesenchymal stem cells, dental mesenchymal stem cells originating from the neural crest have strong neurogenic and immunoregulatory abilities, thus showing promising application and transformation prospects in the field of PNI repair.
- Exosomes play important roles in the repair process of PNI by activating Schwann cells, regulating neuroinflammation, promoting angiogenesis and axonal regeneration, and ameliorating neuropathic pain.
- During the PNI repair process, mitochondria transferred *in vivo* to the site of injury or exogenously transplanted play a role mainly in terms of the energy supply and may also play an immunomodulatory role.
- Cell-derived and tissue-derived extracellular matrices (ECMs) can provide scaffolds for peripheral nerve tissue engineering, and cell-derived ECMs show great potential for clinical translation.

## Background

Peripheral nerve injury (PNI)—induced by mechanical trauma or compressive forces—may precipitate varying degrees of sensorimotor deficits, with clinical manifestations ranging from transient neuralgia to irreversible neuromuscular impairment mediated through Wallerian degeneration pathways [1]. In contemporary clinical practice, surgical intervention continues to serve as the principal therapeutic approach across a spectrum of medical conditions. For peripheral nerve injuries involving transection or short segmental defects, end-to-end suturing is the preferred surgical approach in clinical practice. In cases of PNI with medium to long segmental defects, autologous nerve transplantation is the most commonly used method. Despite being the gold standard in clinical care, this approach has several limitations, including the need for additional surgery, functional loss in the donor area, scarring, and the formation of neuromas. Allografts may serve as a viable clinical option when the nerve defect exceeds 3 cm in length. However, this method is associated with the risk of immune rejection, so immunosuppressive therapy is required. Nonsurgical treatments—such as pharmacological agents, nutritional supplements, electromagnetic stimulation, and cell-based therapies—have shown potential efficacy in the repair of damaged nerves [2]. Consequently, improved repair outcomes are often achieved through a combination of surgical and nonsurgical interventions. Compared with the use of various nerve grafts, facilitating axon regeneration is considered a more advantageous approach. Among these strategies, stem cell therapy has been investigated for its ability to stimulate nerve growth by providing an optimal microenvironment for axonal development [3]. Mesenchymal stem cell (MSC)-based therapy, in particular, is regarded as a highly promising strategy for the management of PNI.

MSCs demonstrate neuroregenerative potential through paracrine secretion of neurotrophins (e.g. brain-derived neurotrophic factor (BDNF) and nerve growth factor (NGF)) during peripheral nerve repair processes, establishing chemotactic gradients that guide axonal sprouting and elongation. Furthermore, MSCs have the potential to differentiate into myelin-forming cell lineages and Schwann-like cells, thereby contributing to nerve repair [4]. Owing to their multifunctional properties, MSCs serve as pivotal seed cells in tissue engineering applications. In this review, the author focuses on several widely studied stem cells and their derivatives that are currently utilized in the treatment of PNI.

## Review

### Mesenchymal stem cells

Stem cells can be categorized into totipotent, pluripotent, and monopotent types based on their differentiation potential, with MSCs falling under the classification of pluripotent stem cells. MSCs are a unique type of stem cell known for their capacity to self-renew, quickly multiply, and transform into various cell types. Because they can modulate the immune system, reduce inflammation, prevent apoptosis, and promote angiogenesis, they are well-suited for regenerative medicine [5]. Current research has identified three primary mechanisms through which MSC therapies exert their effects. First, MSCs possess a unique capacity for multidirectional differentiation, enabling them to replace degenerated and necrotic cells. Second, they secrete anti-inflammatory factors, thereby modulating the immune response within the microenvironment of the injury [6]. Third, MSCs produce a variety of cytokines, including growth factors, cell adhesion factors, and other bioactive molecules that are crucial for promoting tissue regeneration [5, 7]. During peripheral nerve regeneration, extensive cellular communication occurs between MSCs and resident cells. On the one hand, MSCs secrete various neurotrophic factors that act on Schwann cells, stimulating their proliferation and migration [8]. Current studies have found that following peripheral nerve injury, NGF secreted by MSCs binds to the p75 neurotrophic receptor on Schwann cells, which activates intracellular signaling pathways such as the Ras/Raf/MAPK pathway. This activation transitions Schwann cells from a quiescent state to an active repair state, initiating their proliferation and migration to the injury site [9]. Additionally, direct contact between MSCs and Schwann cells influences their function, promoting the expression of nerve regeneration-related genes such as CRYAB, CSPG, and Ki67 [10], as well as the synthesis of proteins such as MBP and MPZ [11]. On the other hand, MSCs exhibit robust immunomodulatory functions, particularly in their interactions with macrophages. Immunoregulatory molecules such as prostaglandin E2 and indoleamine 2,3-dioxygenase are secreted by them, causing macrophages to change from a proinflammatory M1 phenotype to an anti-inflammatory M2 phenotype [12]. MSCs and macrophages work synergistically to clear debris following peripheral nerve injury. While macrophages primarily remove debris through phagocytosis, MSCs enhance this process by secreting factors such as colony-stimulating factor 1 [13]. Given these remarkable properties, stem cell therapy is widely regarded as one of the most promising approaches in regenerative medicine. The transplantation of stem cells holds significant potential for treating peripheral nerve injuries. However, the high degree of heterogeneity among stem cells results in variations in their physiological and applied properties [14], necessitating further research to optimize their therapeutic use.

**Figure 1:**
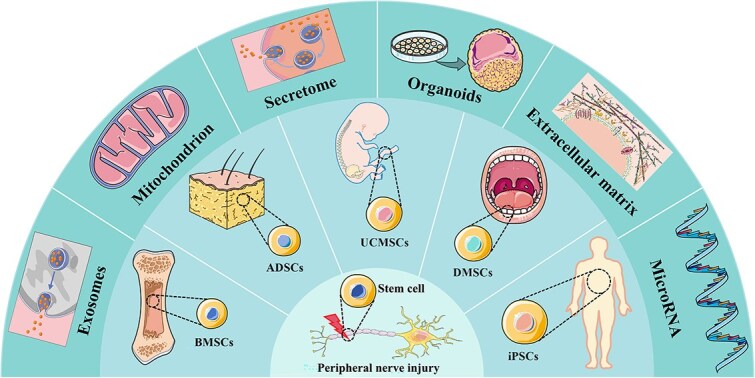
Several common MSC types (mainly BMSCs, ADSCs, UCMSCs, DMSCs, and iPSCs) and their derivatives (mainly exosomes, mitochondria, the secretome, organoids, ECM, and microRNAs) used to promote peripheral nerve regeneration. *BMSCs*, bone marrow mesenchymal stem cells; *ADSCs*, adipose-derived mesenchymal stem cells; *UCMSCs*, umbilical cord mesenchymal stem cells; *DMSCs*, dental mesenchymal stem cells; *iPSCs*, induced pluripotent stem cells. The figure was drawn by the author

Bone marrow mesenchymal stem cells (BMSCs), adipose-derived mesenchymal stem cells (ADMSCs), umbilical cord mesenchymal stem cells (UCMSCs), dental mesenchymal stem cells (DMSCs), and induced pluripotent stem cells (iPSCs) are among the most extensively researched and applied stem cell types. These cells, along with their derivatives—such as exosomes, mitochondria, secretomes, organoids, the extracellular matrix (ECM), and microRNAs—are illustrated in Figure 1. Overall, these cell types demonstrate significant potential for clinical applications in nerve regeneration. Nonetheless, more studies are necessary to identify the best cell type, delivery technique, administration path, mechanism of action, and possible side effects to completely utilize their therapeutic potential.

#### Bone marrow mesenchymal stem cells

Derived from animal bone marrow, bone marrow mesenchymal stem cells (BMSCs) are among the most thoroughly studied cell types in relation to peripheral nerve injury [15]. BMSCs exhibit immunomodulatory properties, low immunogenicity, a homing effect, and the ability to secrete bioactive substances, all of which have been shown to promote tissue repair [16–18]. In response to specific environmental stimuli, these pluripotent stem cells can differentiate into various cell types, including mesenchymal cells (such as bone, adipose, and cartilage cells) and nonmesenchymal cells (such as neurons and Schwann-like cells) [5, 11, 19]. Li *et al.* demonstrated that integrating BMSCs with acellularized nerve grafts (ANAs) boosted immunomodulatory effects, evidenced by a notable rise in regulatory T cells (Tregs), which facilitated the recovery of sciatic nerve function. Furthermore, the therapeutic effects of BMSCs were similar to those of adipose-derived stem cells (ADSCs) in treating sciatic nerve injuries [20]. BMSC-derived extracellular vesicles have been demonstrated to enhance angiogenesis at injury locations, boost axon and myelin development, and heal sciatic nerve injuries in rats, possibly by regulating messenger RNAs (mRNAs) [21, 22]. BMSCs secrete a variety of neurotrophic factors, such as NGF, BDNF, Glial-cell-line-derived neurotrophic factor (GDNF), and Ciliary neurotrophic factor (CNTF), while also depositing ECM components [23]. They have been shown to downregulate proinflammatory cytokines, including IL-1β, IL-6, and TNF-α, while upregulating TGF-β, thereby inhibiting inflammatory responses, modulating the immune microenvironment, reducing neuropathic pain (NP), and promoting nerve repair [24, 25]. Furthermore, Zhong *et al*. reported that BMSC transplantation alleviates pain by upregulating the PI3K/AKT pathway, downregulating the NF-κB pathway, and releasing GDNF, among other mechanisms [26]. The application of BMSCs is constrained by worries about their invasiveness and ethical debates, despite their potential benefits.

#### Adipose-derived mesenchymal stem cells

Isolated from the stromal vascular fraction of adipose tissue, ADSCs are considered an excellent source of MSCs due to their high availability, easy accessibility, and powerful immunomodulatory functions. Notably, their therapeutic potential remains unaffected by the donor age or anatomical harvesting site while maintaining stable phenotypic characteristics and long-term plasticity *in vitro* [27]. ADSCs possess unique practical benefits over bone marrow-derived mesenchymal stem cells (BMSCs), as they can be routinely harvested via minimally invasive liposuction while achieving higher yields, thereby improving their translational potential in clinical settings [28, 29]. These unique properties position ADSCs as a pivotal cellular resource for tissue engineering and neural regeneration applications. The neuroregenerative capacity of ADSCs stems from their Schwann cell-like differentiation potential and neurotrophic factor secretion. Soto *et al*. demonstrated that magnetically guided ADSC delivery significantly increased myelinated fiber density after sciatic nerve injury, which was correlated with improved nerve conduction velocity and motor function recovery [30]. Complementary findings reported by Rodríguez Sánchez *et al*. revealed accelerated pro-regenerative effects within 2–4 weeks after the transplantation of canine ADSCs in rodent sciatic nerve crush models [31]. Liu *et al*. further extended these observations to chronic injury settings, showing an ADSC-mediated reduction in dorsal root ganglion inflammation alongside enhanced axonal regrowth and remyelination [32]. Despite these promising results, post-transplantation survival remains a critical challenge. Innovative strategies employing collagen I hydrogel scaffolds significantly improved fiber organization, angiogenic activity, and neurotrophic factor release in the dorsal root ganglia when these scaffolds were used as ADSC carriers [33]. Microcarrier technology has shown particular promise, with polylysine surface modifications increasing cellular adhesion [34]. Zhu *et al*. achieved superior outcomes by incorporating ADSC-loaded microcarriers into chitosan nerve conduits, which synergistically promoted cell viability, proliferation, and directional migration for optimized nerve repair [35]. In addition to scaffold-based strategies, supplementing ADSC culture media with platelet lysates has been shown to enhance their neurotrophic properties [36, 37]. The adipose-derived vascular stromal fraction, a cell population with stem cell-like characteristics, has also been employed as a cellular adjunct to autologous fat grafting. This approach promotes angiogenesis, reduces inflammation, and minimizes fibrosis, thereby improving the survival and therapeutic outcomes of ADSC transplantation [38]. Furthermore, studies have identified the roles of FGF9 and Let-7a-5p in facilitating ADSC differentiation into Schwann-like cells, highlighting potential molecular targets to optimize their therapeutic potential [39, 40]. Collectively, ADSC-based therapies exert multifaceted neurorestorative effects through axonal regeneration, remyelination, and immunomodulation. When integrated with advanced biomaterial carriers, bioactive supplements, and molecular adjuvants, ADSCs constitute a robust cellular platform for next-generation neural tissue engineering strategies.

#### Umbilical cord mesenchymal stem cells

Derived from the umbilical cords of newborns, UCMSCs provide a safe and easily obtainable source of stem cells with notable therapeutic benefits. Compared with MSCs derived from adult tissues, UCMSCs exhibit a superior proliferative capacity, enhanced differentiation potential, and more potent immunomodulatory properties [41, 42]. Bojanic *et al*. conducted a comprehensive review of UCMSC applications in peripheral nerve injuries and found that UCMSC transplantation significantly increased the axonal number and density, along with sciatic nerve functional indices, compared with those in control groups in animal models [43]. The therapeutic potential of UCMSCs has been further enhanced through cellular reprogramming approaches. The differentiation of UCMSCs into Schwann-like cells by Lin *et al*. was successful, boosting the expression of proangiogenic and neurotrophic factors and thus improving their therapeutic effectiveness [44]. Parallel research findings indicate that the combined administration of Schwann-like cells differentiated from UCMSCs and decellularized nerve scaffolds in neural regeneration therapy elicits significantly higher BDNF and GDNF levels at lesion sites than direct UCMSC implantation with decellularized grafts [42]. Guan *et al*. also developed dual-bionic nerve regeneration materials using UCMSCs to provide adequate support for axonal regeneration and angiogenic processes [45]. Zhang *et al*. observed that platelet-rich plasma exosomes maintain the stemness of UCMSCs, increase their activity, and attenuate their apoptosis under stress. In a laboratory setting, MSCs encourage the growth of Schwann cells and axons in the dorsal root ganglion [46]. Researchers have proposed that the immunomodulatory and paracrine effects of UCMSCs are more important than their differentiation capacity in their therapeutic application for PNI [4]. Compared with the control, the cotransplantation of UCMSCs with erythropoietin increased the number of axons and neurofilament (NF) density [47]. Experimental investigations by Zhu *et al*. demonstrated that exosomes derived from hypoxia-preconditioned UCMSCs markedly enhanced the migratory recruitment of Schwann cells in peripheral nerve lesions, with concomitant *in vitro* validation of their therapeutic efficacy in neural regeneration. Notably, cellular internalization assays confirmed the mechanistic uptake of these exosomes by Schwann cells during regenerative processes [48]. Consequently, UCMSC-derived exosomes are predicted to be beneficial for NP treatment after being validated in large animal and clinical trials. UCMSCs can be employed as stem cell therapies to promote pro-neural axonal and myelin growth in the repair of PNI. Certain exogenous factors—such as platelet-rich plasma and neural grafting materials—can enhance the efficacy of UCMSC therapy.

#### Dental-derived mesenchymal stem cells

Through various mechanisms such as paracrine effects, vascular and synaptic regeneration, immune system modulation, and apoptosis inhibition, DMSCs may be crucial in treating neurological conditions. The isolation of dental stem cells from the pulp of permanent teeth was first achieved by Gronthos in 2002, who named them dental pulp stem cells (DPSCs), indicating that dental tissues could potentially be a source of stem cells. The taxonomic delineation of odontogenic stem cells is fundamentally contingent upon their distinctive anatomical derivation. The following subpopulations have been identified: DPSCs, periodontal stem cells (PDLSCs), deciduous stem cells (SHEDs), and gingival mesenchymal stem cells (GMSCs). Furthermore, they are capable of differentiating into various cell lineages, including osteoblasts, adipocytes, chondrocytes, myoblasts, and neuronal cells, among others. DPSCs, PDLSCs, and SHEDs all originate in the neural crest, which is a significant distinction from the source of BMSCs, which originate from the mesoderm [49]. Additionally, DPSCs demonstrate clonogenicity and a robust cell proliferation capacity. They are less susceptible to tumor formation than other MSCs are. Comparative analysis reveals distinct functional profiles among dental stem cell populations: DPSCs demonstrate pronounced neurogenic potential through modulation of endogenous progenitor cell recruitment dynamics and facilitation of neural lineage differentiation pathways [50]. SHEDs exhibit superior proliferative vigor and metabolic competence relative to BMSCs. Notably, DPSCs demonstrate a significant retardation in replicative senescence compared to UCMSCs under *in vitro* culture conditions. DPSCs and SHEDs exert beneficial effects on vascular regeneration, neurotrophic factor secretion, and immune regulation while also exhibiting the capacity to undergo directed migration to injured regions. Furthermore, these cells can be readily autologously transplanted.

Compared with the majority of MSCs, DMSCs derived from the neural crest display enhanced proliferative and neural differentiation capabilities, exhibit remarkable anti-inflammatory and immunomodulatory effects, and can be harvested in a manner that circumvents invasive surgical procedures while facilitating expeditious gingival tissue healing without scarring. Despite their disparate origins, they all exhibit analogous properties, including self-renewal, multidirectional differentiation, immunomodulation, and the expression of MSC surface markers [49]. Furthermore, DMSCs exhibit enhanced proliferative and neural differentiation capabilities, which are advantageous for nerve regeneration. Current research findings demonstrate that DMSCs exhibit superior suitability for translational implementation in regenerative medicine when compared to alternative stem cell sources [51–53]. DMSCs are more accessible, demonstrate robust differentiation potential, and exhibit high plasticity. Consequently, these attributes render the transplantation of DMSCs a feasible approach for the treatment of neurological diseases [54]. MSCs can be isolated from dental tissue and obtained from various sources, including the pulp, periodontium, deciduous teeth, gingiva, and papilla. Compared with BMSCs, DPSCs have been shown to possess enhanced capabilities for neural regeneration. Consequently, DPSCs have recently emerged as a subject of considerable interest within the scientific community. PDLSCs are promising sources of cells with potential therapeutic applications in neural repair. PDLSCs are capable of releasing neurotrophic factors, which facilitate the formation of myelin and the regeneration of neurons. Due to their accessibility, biological compatibility, and ethical acceptability, DMSCs have emerged as a significant source of cell therapy in nerve repair [55]. The less invasive nature of the acquisition pathway has been shown to confer advantages to DMSCs over other MSCs in the context of tissue repair and regeneration [56].

Emerging experimental evidence reveals that GMSC-differentiated Schwann-like cells possess dual-functional characteristics encompassing neurotrophic support and macrophage polarization regulation. Mechanistically, these biological attributes demonstrate marked therapeutic efficacy in potentiating axonal regrowth and functional neural restoration in rodent sciatic nerve injury models [57]. In response to inflammatory stimuli, GMSCs secrete a substantial quantity of soluble mediators, thereby exerting immunosuppressive effects. Exosomes derived from TNF-α-interacting GMSCs have been shown to inhibit microglial activation and attenuate the intensity of the neuroinflammatory response, potentially via the meg3/miR-21a-5p axis [58].

#### Induced pluripotent stem cells

iPSCs, formally defined as reprogrammed pluripotent stem cells, constitute a specialized class of pluripotent cells generated through defined transcriptional factor-mediated epigenetic reprogramming of terminally differentiated somatic cells, ultimately possessing molecular and functional equivalency to embryonic stem cells. This genetically engineered cell lineage demonstrates embryonic stem cell-equivalent totipotency through CRISPR/Cas9-mediated genetic modification, exhibiting congruent molecular signatures in cellular morphology, transcriptional profiles, proteomic expression patterns, and multilineage differentiation capacity that meet stringent equivalence criteria to their embryonic counterparts, thereby achieving functional equivalency in regenerative therapeutic applications. Its potential applications are nearly identical to those of embryonic stem cells. iPSCs offer multiple advantages. Primarily, they can be derived from a wide range of sources and can be artificially synthesized without ethical constraints. Second, iPSCs exhibit a robust regenerative differentiation capacity, which provides a novel avenue for advancement in the broader field of stem cell therapy and regenerative medicine research [59].

Oncomodulin (OCM) is a myeloid-derived growth factor that has been reported to promote axon regeneration following optic nerve injury or peripheral nerve injury. Xie *et al*. identified ArmC10 as a receptor with high affinity for OCM and showed that the deletion of ArmC10 inhibits axon regeneration and that ArmC10 is highly expressed in sensory neurons derived from iPSCs. Therefore, iPSCs may provide important intermediate mediators for nerve repair [60]. Bioengineered neural fascicles can be systematically fabricated from iPSC-differentiated neural organoids through innovative microfluidic bioprinting platforms, demonstrating significant therapeutic potential for peripheral nerve deficits by mechanistically replicating native neural architecture while maintaining functional axonal guidance capabilities [61]. iPSCs demonstrate remarkable versatility within regenerative medicine frameworks targeting peripheral nerve repair. Through directed differentiation protocols employing defined culture conditions with lineage-specific morphogens, these pluripotent cells can be systematically converted into functional neural lineages encompassing sensory neurons, radial glial progenitors, multipotent neural stem cells, supportive neuroglia, and astrocytic populations, enabling precise temporal regulation of cellular maturation for optimal functional integration in therapeutic contexts. iPSCs can be used for PNI therapy alone or together with Schwann cells and neuronal cells after bioprinting with scaffolding materials [62]. Pan *et al*. reprogrammed human fibroblasts into iPSCs through gene editing, differentiated them into mature neurons and Schwann cells *in vitro*, and observed that exosomes extracted from iPSCs can be taken up by Schwann cells, promoting Schwann cell proliferation [63]. Tam *et al*. developed a neuroregenerative paradigm employing human iPSC-differentiated nociceptive neurons to orchestrate the transdifferentiation of hBMSC-derived neurospheres into functional Schwann cell phenotypes. These engineered cellular constructs were surgically engrafted within biodegradable nerve guidance conduits at neural lesion sites, with histomorphometric validation confirming their capacity to promote motor pathway reconstruction through enhanced axonal myelination and functional reinnervation metrics in post-traumatic recovery models [64]. Mitsuzawa *et al*. surgically engrafted a 3D-bioprinted neural guidance conduit engineered from iPSC-derived mesenchymal stromal cells into rodent sciatic nerve defects, with parallel microcatheter-mediated delivery of cellular constructs into the injury microenvironment. Histopathological analyses and functional assessments mechanistically demonstrated the iMSC-mediated dual regenerative capacity for vascular network reconstruction and axonal regrowth metrics through paracrine-mediated trophic modulation [65]. Schwann cell precursors reprogrammed from human dermal fibroblasts exhibit potent neurotrophic secretory capacity (GDNF, NGF, BDNF, and NT-3) and undergo terminal differentiation into mature Schwann cells, enabling functional restoration of sciatic nerve motor pathways [66]. Manion *et al*. demonstrated that GABAergic neurons derived from iPSCs significantly and safely alleviated NP induced by peripheral nerve injury in mice for long periods [67]. Furthermore, human embryonic stem cells (hESCs) are functionally equivalent to iPSC-derived Schwann cells, with few proteomic differences and some transcriptomic differences between them. In addition, Schwann cells secrete a variety of neurotrophic factors that promote axonal regeneration and direct nerve regeneration pathways after nerve injury, and these cells create a favorable environment for nerve regeneration via the phagocytosis of myelin debris [68]. For future treatments, we need to optimize cell therapy protocols.

The choice of cell type as well as the mode and quantity of cell transplant delivery will affect the final therapeutic effect. In-depth studies of the mechanisms of action, modulation of signaling pathways, and cell–cell interactions are needed. The challenges include resolving the quality control issues of transplanted cells, immune rejection, and ethical issues. In clinical translation, a greater need is to identify accurate molecular mechanisms and assess their efficacy. The mechanisms of action, signaling pathways, and advantages and disadvantages of different MSCs for the treatment of nerve injury are summarized in Table 1.

**Table 1:** The therapeutic utilization of common stem cell types for peripheral nerve regeneration requires systematic analysis through cellular origin, signaling axis, therapeutic merits/limitations, and molecular effector pathways

Source	Signaling pathways/cytokines	Advantages	Disadvantages	Outcomes/references
BMSCs	TGF-β, BDNF, GDNF, PI3K/AKT, and NF-κB	Widely sourced, multilineage differentiation, homing mechanism, and secretes multiple bioactive substances	Invasive acquisition, ethical controversy	Immunomodulation, increased number of Tregs [20] Promotes angiogenesis and axonal and myelin regeneration [22] Modulates inflammation and relieves NP [25] Suppresses inflammation, neurotrophic, and relieves NP [26]
ADSCs	NGF, FGF9, and Let-7a-5p	Widely sourced, easy acquisition, independent of the tissue source site and donor age, and long-term stability and plasticity *in vitro*	Limited differentiation, ability, and additional surgery required	Promote the growth of myelinated nerve fibers [30] Attenuate leukocytic infiltration while enhancing axonal remyelination [32] Promote dorsal root ganglion growth and angiogenesis [33] Enhancement of ADSC activity and proliferation through combination with scaffolds [34] Promote ADSC differentiation into Schwann cells [39]
UCMSCs	BDNF, GDNF, VEGF, FGF-2, IL-10, and c-Fos	Easy acquisition and harmless and better capacity for cellular activity, proliferation, differentiation, and immunomodulation	Expensive to keep for long periods of time	Increased axon number and density [43] Promote angiogenesis, neurotrophic, and differentiation into Schwann cells [44] Axonal regeneration and angiogenesis [45] Stimulate Schwann cell mitogenesis and dorsal root ganglion (DRG) neuritogenesis [46]
DMSCs	BDNF, GDNF, IL-10, MEG3, and miR-21a-5p	Greater capacity for neural spectrum differentiation, low invasiveness, and targeted migration capacity	Varied and unstable sources	Promote endogenous stem cell recruitment and neural crest cell generation [50] Neurotrophic, immune regulation, and promote nerve regeneration [57] Inflammation suppression and immunomodulation [58]
iPSCs	ArmC10/OCM and organoids	Widely sourced, synthetic and editable, exclusion of immune rejection, and no ethical controversy	Inefficient induction, risk of retention, and expensive for clinical applications	Organoid composition of artificial axon bundles [61] Differentiate into neurons and glial cells [62] Promote angiogenesis and nerve regeneration [65] Differentiate into Schwann precursor cells [66] Differentiate into GABAergic neurons to relieve NP [67]

#### Preclinical studies

Guo *et al*. treated 91 spinal cord injury (SCI) patients with autologous BMSC transplantation and reported that 85 of them experienced partial improvements in motor, sensory, and neurophysiological functions, whereas six patients with ineffective treatment had complete chronic SCI and a duration of injury of more than 5 years, which was presumed to be due to the degeneration and necrosis of nerve fibers in the late stage of SCI, glial scarring, and spinal cord cavities, which are unfavorable for SCI repair [69]. However, Mendonca *et al*. performed intralesional BMSC transplantation in patients with chronic traumatic thoracic or lumbar medullary injuries (>6 months) and reported a good neurological improvement, suggesting that BMSCs can be used to treat patients with chronic complete SCI. Experimental data reveal that BMSC-mediated therapeutic efficacy exhibits temporal dependency relative to spinal cord injury progression windows [70]. Dai *et al*. enrolled 40 chronic complete cervical SCI patients in a randomized controlled trial, allocating participants into therapeutic and control cohorts [71]. The intervention cohort received localized BMSC administration at lesion sites, whereas the non-transplant cohort underwent standard care. Notably, therapeutic recipients demonstrated significant neurological recovery (motor/sensory modalities) with reduced urinary retention volumes. Contrastingly, the control group showed neither functional improvement nor tumorigenic events, though neoplastic manifestations emerged in 6-month post-intervention evaluations of treated subjects.

Combinatorial cellular therapy with autologous MSCs and Schwann cells demonstrated significant neurological recovery in 106 complete SCI patients during therapeutic intervention and follow-up monitoring. Targeted intrathecal delivery of BMSC-SC combinations enhanced urodynamic metrics while reducing urinary incontinence incidence and elevating quality of life (QoL) parameters in neurogenic bladder cases [72]. Clinical research demonstrated autologous Schwann cell and bone marrow-derived MSC (BM-MSC) transplantation during the subacute SCI phase significantly enhanced sensorineural functional outcomes in patients [73]. Clinical investigation into intrathecal ADMSC administration for traumatic spinal cord injury (TSCI) demonstrated american spinal injury association impairment scale (AIS) grade elevation in seven patients during post-intervention assessments compared to baseline injection metrics [74]. In 2023, China’s Guangdong Provincial Hospital Association released the group standard “Clinical Technical Specification for the Treatment of Traumatic Spinal Cord Injury with Human MSCs Transplanted in the Subarachnoid Space” to better accommodate the development of cell therapy.

The clinical trial demonstrated favorable safety profiles for both MSC variants. Subjects exhibited statistically significant enhancements in sensorimotor domains. Furthermore, anal sphincter control improvements with enhanced deep pressure perception were documented across cohorts, correlating with enhanced QoL metrics. Significantly, two Group B participants achieved complete bladder autonomy, eliminating catheterization dependence [75]. Subsequent clinical investigation demonstrated favorable safety–efficacy profiles of autologous MSC administration in 13 SCI cases [76]. In terms of challenges, we need to address the standardization of stem cell sources, long-term survival after transplantation, etc.

### Exosomes

Contemporary research establishes MSCs as mediating therapeutic outcomes through exosomal secretion. These nanoscale extracellular vesicles (30–150 nm diameter) feature a lipid bilayer envelope. Their biogenesis initiates with plasma membrane invagination forming early endosomes, progressing through endosomal sorting complex required for transport-mediated maturation where intraluminal vesicle budding occurs. Subsequent membrane fusion enables exocytic secretion of these vesicles. Crucially, exosomes transport diverse cargo including regulatory RNAs, functional proteins, lipid signaling molecules, and bioactive metabolites [77, 78].

**Figure 2:**
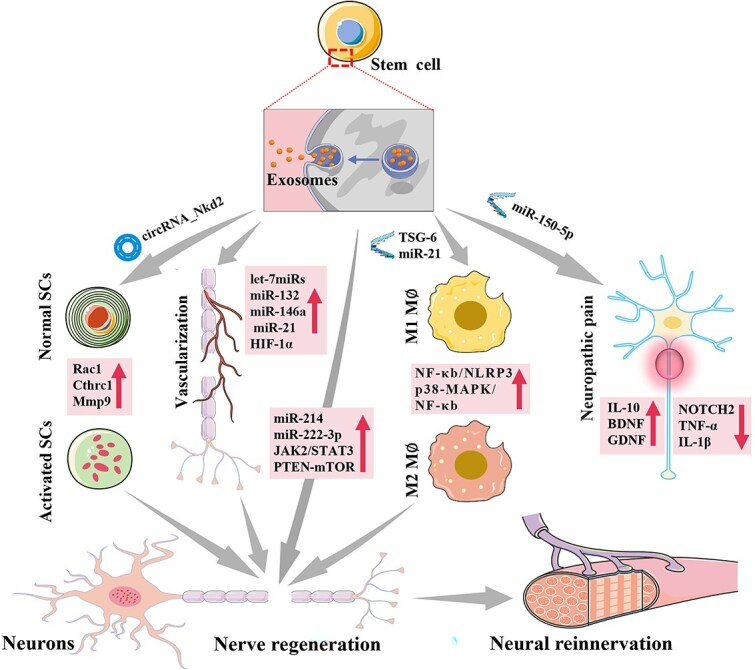
Stem cell-derived extracellular vesicles can directly act on neurons and axons at the injury site to promote peripheral nerve regeneration. Moreover, they can promote peripheral nerve regeneration by stimulating Schwann cells to dedifferentiate into repair-type Schwann cells, inducing angiogenesis, stimulating proinflammatory macrophages to transform into anti-inflammatory macrophages, alleviating NP, and ultimately restoring neural reinnervation of the target organ. The figure was drawn by the author

Exosomes derived from stem cells present several advantages over MSCs, including their smaller size, good biocompatibility, low toxicity, stability, and ability to act as molecular carriers. These vesicles mediate intercellular signaling and modulate tissue-level homeostatic regulation [79]. Consequently, exosomes have advanced as promising therapeutic modalities within regenerative medicine frameworks [80, 81]. Exosomes have been shown to exert a range of beneficial effects following nerve injury, including immunomodulatory, anti-inflammatory, antiapoptotic, and pro-vascular and axonal regenerative effects, which collectively contribute to functional recovery. Stem cell paracrine signaling mediates microenvironmental regulation through transplanted cellular secretomes, where neurotrophic factors orchestrate neuronal viability and lineage commitment [82]. Consequently, we reviewed the literature on the therapeutic role of MSC-derived exosomes in PNI. Figure 2 shows the multiple mechanisms by which extracellular vesicles derived from stem cells promote peripheral nerve regeneration and relieve NP.

#### Nerve repair facilitators

##### Axonal regeneration

The most intuitive method for restoring nerve integrity is axonal growth, which represents the primary objective of numerous therapeutic strategies for nerve injury. Emerging research establishes MSC-derived exosomes as innovative nanovesicle-based gene delivery systems that activate nerve regeneration-related pathways and promote nerve regeneration through the delivery of microRNAs (miRs) to target cells, thereby regulating gene expression [83, 84]. Low-intensity ultrasound-primed Schwann cell-derived exosomes enriched with pluripotent miRs cargos selectively activate the PI3K-Akt-FoxO axis, upregulating PI3K/Akt/FoxO phosphorylation cascades within ganglionic neurons, thereby potentiating neural regenerative outcomes [85]. Analogously, MSC-derived exosomes engineered for miR-214 overexpression demonstrate JAK2/STAT3 pathway activation through PTEN-targeted signaling modulation [86]. PTEN operates as a mechanistic nexus within the mTOR signaling axis, with the PTEN-mTOR regulatory cascade established as a critical determinant of axonal regenerative capacity [87]. In optic nerve injury, Sang *et al*. demonstrated that miR-222-3p—which is delivered by exosomes derived from UCMSCs—can target the mTORC signaling pathway [88]. The aforementioned studies present a promising class of therapeutic methods for nerve injury. Additionally, exosomes can be internalized by neuronal cells, where they perform regulatory functions. Some studies have also described the efficacy of a combined approach in which nerve conduits and exosomes are utilized to optimize the promotion of axonal growth function [89, 90]. Given that exosomes contain trophic factors, they are capable of providing a superior microenvironment for nerve regeneration when used in conjunction with nerve conduit grafts [91]. Extracted exosomes can indirectly increase cell viability and stemness by acting on other MSCs to increase their proliferation and antiapoptotic effects, thus enabling MSCs to better promote peripheral nerve repair and regeneration [46]. Therefore, the regulation of gene expression, cytokines, and trophic factors, or the combination with nerve conduits through miRs, can promote nerve regeneration. In addition, an extensively investigated aspect is the engineering of exosomes [92], whereby MSCs are manipulated by the knockdown or overexpression of genes, stimulated by inflammatory factors, and preacclimated to hypoxic culture to functionalize the exosomes for tissue repair.

##### Schwann cell activation

Post-exosomal internalization, Schwann cells acquire augmented functional competence. Hu *et al*. demonstrated efficient cellular uptake of both hAMSC-exos and SCLC-exos by Schwann cells, with SCLC-exos displaying superior efficacy in stimulating Schwann cell mitogenesis and chemotaxis. Post-internalization, these cells orchestrate myelination via elevated cytokine secretion while facilitating concerted axonal and vascular regeneration [93]. Additionally, Wang *et al*. demonstrated that exosomes from hypoxic cultures of MSCs promote the proliferation, migration, and paracrine secretory functions of SCs and that these functions are promoted more effectively through the high expression of circRNA_Nkd2, which, in turn, promotes repair after facial nerve injury [91]. *In vitro* analyses demonstrated that MSC-exos potentiate Schwann cell mitogenesis, chemotaxis, myelination, and neurotrophic secretion via transcriptional upregulation of Rac1, Cthrc1, and Mmp9 [94]. Huang *et al*. elucidated that endothelial-derived exosomes undergo Schwann cell internalization to sustain proliferative activity and paracrine functionality. This regenerative modulation is mechanistically governed by PI3K/Akt/PTEN signaling axis activation, stabilizing Schwann cells in a pro-regenerative phenotypic state [95]. Chen *et al*. elucidated that hAMSC-induced Schwann-like cell-derived exosomes potentiate Schwann cell mitogenesis, chemotaxis, and functional activation in sciatic nerve injury models via the SOX2/FN1 signaling axis [96]. Zhang *et al*. elucidated that PRP-derived exosomes cocultured with UC-MSCs enhance Schwann cell mitogenesis and DRG neuritogenesis via neurotrophic paracrine signaling [46]. Comparatively assessing neural stem cell-, fibroblast-, and Schwann cell-derived exosomal capacities to induce BMSC-to-Schwann cell differentiation, their findings demonstrated that SC-exos exerted superior efficacy over NSC-exos, with both outperforming fibroblast-exos [97, 98]. Experimental evidence demonstrates that exosomes from cutaneous Schwann progenitor cells potentiate Schwann cell activation and induce sensory/motor neuritogenesis, with therapeutic efficacy validated in canine sciatic injury models [99–101]. Exosomes enhance Schwann cell-mediated peripheral nerve repair by (i) enhancing proliferative, migratory, and survival capacities; (ii) reprogramming Schwann cells into regenerative phenotypes during neural restoration; and (iii) paracrine-mediated activation through stimulating stem cell-derived Schwann cell differentiation. Notably, exosomes exhibit source-dependent heterogeneity in both therapeutic magnitude and mechanistic pathways targeting Schwann cell functionality.

##### Neuroangiogenesis

The development of the nervous system is supported by the formation of blood vessels. In addition to supplying oxygen and nutrients to peripheral neurons, which require an energy source, blood vessels also function as active signaling systems [102]. The formation and perfusion of vessels are highly dependent on the function and regeneration of nerves. Researchers have identified a robust interconnection between nerve regeneration and blood vessel formation subsequent to injury [103]. The vascular system can provide migratory pathways for Schwann cells, and vascular endothelial cells secrete a variety of bioactivators that favor axonal growth [103, 104]. In clinical practice, the use of nerve grafts with incorporated blood vessels has been shown to provide an optimal environment for the regeneration of nerves, thereby facilitating the rapid recovery of nerve function [105]. Therefore, the reconstruction of the vascular network following peripheral nerve injury is imperative.

A number of recent studies have revealed that exosomes can act as regulatory substances to promote vascular regeneration. Gong *et al*. found that exosomes obtained from GATA-4-overexpressing MSCs increased the proangiogenic delivery of let-7 microRNAs (miRs) that target platelet reactive protein-1 in endothelial cells [106]. Heo *et al*. elucidated that ADMSC-derived exosomes deliver miR-132/146a cargo to transcriptionally activate proangiogenic programs, thereby stimulating mitogenic activity and vasculogenic tubulogenesis in HUVEC cultures [107]. Zhang *et al*. elucidated that hUCMSC-derived exosomes stimulate endothelial progenitor cell mitogenic, migratory, and angiogenic capacities; engineered exosomal miR-21 cargo was further shown to orchestrate neovascularization mechanistically mediated through NOTCH1/DLL4 axis activation [108]. Phelps *et al*. elucidated that MSC-derived exosomes orchestrate cerebral microvasculature neovascularization via angiogenic differentiation pathways, concomitant with neurological functional recovery [109]. Sun *et al*. demonstrated exosomal HIF-1α overexpression *in vitro* restored angiogenic/migratory/proliferative capacities of compromised umbilical vein endothelial cells while upregulating proangiogenic mediators *in vivo*. These exosomes exert a cardioprotective effect by promoting neural angiogenesis and inhibiting fibrosis [110]. Therefore, a reasonable inference is that exosomes promote peripheral nerve regeneration by enhancing angiogenesis [93].

#### Adverse effects associated with nerve injury

##### Regulation of neuroinflammation

MSCs possess robust immunomodulatory capabilities, and their secreted exosomes have been shown to exert similar effects. Following the onset of peripheral nerve injury, Schwann cells phagocytose and remove myelin debris, secrete proinflammatory cytokines, and recruit immune cells when Wallerian degeneration occurs. First, the earliest cells recruited to the site of injury are granulocytes and neutrophils, which can prevent secondary infections from bacteria and other microorganisms. Eosinophils may also participate in tissue repair processes, as they can release various mediators that contribute to tissue remodeling [111]. Mast cells can release bioactive substances such as histamine, increase vascular permeability, and enable nutrients and immune cells in the blood to reach the site of injury. Second, recruited macrophages can also engulf and clear the myelin debris produced by damaged nerve fibers. Infiltrating T-cell subsets including Tregs mediate immunomodulation through pleiotropic cytokine secretion, effectively mitigating secondary neuroinflammation-induced neural tissue degeneration [112]. Finally, cells at the site of injury release inflammatory mediators such as IL-1β and TNF-α, which activate surrounding neural stem cells and progenitor cells to proliferate and differentiate into neurons or glial cells [6]. Hyperinflammatory states trigger leukocyte liberation of cytotoxic mediators (ROS/NO, reactive oxygen species/nitric oxide) that mediate neurotoxicity through direct neuronal/glial structural damage, culminating in apoptotic cascades [113]. If the inflammatory response is not controlled in a timely manner, chronic inflammation may occur. Persistent inflammatory processes demonstrate a pivotal association with NP pathogenesis [114]. Exosomes potentiate neural regenerative niches via M1-to-M2 macrophage polarization [115]. Exosomes derived from lipopolysaccharide-pretreated MSCs possess robust immunomodulatory and anti-inflammatory properties. These exosomes have been shown to facilitate the transformation of proinflammatory macrophages (M1-type) into proregenerative macrophages (M2-type). Complementary mechanistic investigations elucidate exosomal suppression of the NF-κB/NLRP3 axis via TSG-6 cargo delivery [116]. Moreover, Xue *et al*. demonstrated that MSC-derived exosomes orchestrate M2 macrophage polarization and mitigate inflammatory pathogenesis through the p38-MAPK/NF-κB signaling axis [117]. Within the central nervous system (CNS), exosomal miR-21 deficiency orchestrates neuroprotective effects by suppressing neuroinflammatory responses, maintaining BBB integrity, inhibiting apoptotic cascades, and facilitating functional neural recovery post-brain injury [118].

##### Neuropathic pain

NP arises from neural lesions or aberrant neural signaling, manifesting as chronic pain. Peripheral nerve trauma (PNI) inducing sensory fiber lesions triggers NP, substantially compromising patient quality of life metrics. At present, no specific pharmacological agent is capable of completely treating NP. Some studies have indicated the potential existence of an association between NP and low-grade inflammation [119]. Exosomes possess immunomodulatory and anti-inflammatory properties, as evidenced by a number of recent studies in this field. Buchheit *et al*. observed that conditioned serum exosomes were highly efficacious at alleviating NP associated with paclitaxel-induced peripheral neuropathy. Furthermore, the analgesic effect was sustained over an extended period, which was attributed to the blockade of chemotherapy-induced neuropathy. The conditioned serum exosomes induced an anti-inflammatory response in glial cells, with significantly increased anti-inflammatory factor concentrations [120]. Shiue *et al*. concluded that the analgesic effect of exosomes was related to neurons and glial cells, and the anti-inflammatory and neurotrophic effects of exosomes were identified through the detection of relevant indices [121]. With respect to the therapeutic mechanisms involved, studies have indicated that microRNAs play a significant role. For example, Li *et al*. demonstrated that miR-150-5p in exosomes derived from BMSCs alleviated mechanically abnormal pain by targeting NOTCH2 in microglia [122].

### Mitochondrial

Mitochondria serve as the principal intracellular organelles for cellular energy production. Their structural architecture features a double-membrane system organized into four distinct compartments: outer membrane, inner membrane, intermembrane space, and mitochondrial matrix. The inner membrane is populated with a multitude of respiratory chain complexes, which are instrumental in the oxidative phosphorylation process and facilitate the conversion of nutrients into adenosine triphosphate (ATP), thereby supplying the cell with energy [123, 124]. Contemporary research has elucidated mitochondrial homeostatic regulation, quality control mechanisms, and interorganellar crosstalk as a novel research focus [125, 126]. A substantial body of evidence attests to the intimate connection between mitochondrial function and nervous system function. During the regeneration of nerve axons, growth cones, ion channels, and ion pumps rely on a substantial energy supply to perform their functions [127]. Consequently, mitochondria play a pivotal role in recovery from nerve injury [128]. Building upon contemporary scientific progress, we systematically explored mitochondrial therapeutic interventions for peripheral neural regeneration.

#### Mitochondrial transfer

**Figure 3:**
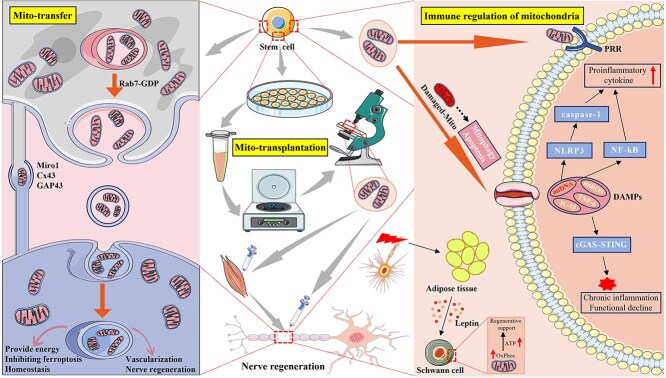
Stem cell-derived mitochondria mediate neural regeneration via molecular mechanisms including mitochondrial transfer, transplantation, and immunomodulatory pathways. Mitochondrial transfer primarily operates through intercellular proximity, utilizing dual transfer modalities: TNTs (Cx43/GAP43/Rho-GTPase Miro1-regulated) and Rab7-GDP-mediated vesicular transport. Mitochondrial transfer enhances peripheral nerve repair via bioenergetic support, ferroptosis suppression, neovascularization induction, and homeostatic microenvironment modulation. Mitochondrial transplantation involves extracting exosomes from stem cells through artificial techniques. After identification and concentration, they can be used to target muscles or directly for nerves at the injury site. In terms of immune regulation, mitochondria can enter recipient cells through PRRs; mitochondria can also activate the innate immune response of cells by releasing mtDNA into the cytoplasm, increasing the levels of proinflammatory cytokines, and causing chronic inflammation and functional decline through different signaling pathways. In addition, adipose tissue at the site of nerve injury can act on Schwann cells by secreting leptin, thereby increasing the ATP content inside the cells to support regeneration damage-associated molecular patterns. The figure was drawn by the author

Intercellular mitochondrial transfer from MSCs modulates key cellular functions in recipient cells, encompassing proliferation, migration, differentiation, metabolic reprogramming, and phenotypic plasticity. The main pathways for the mitochondrial transfer of MSCs include tunneling nanotubes (TNTs) (Cx43, GAP43, and Rho-GTPase Miro1-dependent pathways), extracellular vesicles (Rab7-GDP-dependent pathway), gap junctions, and cell fusion. Furthermore, cells are also capable of ingesting free mitochondria that have been released by MSCs [129]. Following PNI, the vascular system at the site of injury is disrupted, and the formation of new blood vessels provides pathways for the transportation of nutrients, which are essential for the reconstruction and functional recovery of the nerve after injury. In a recent study, Lin *et al*. observed that mitochondria from MSCs were transferred to endothelial cells by TNTs, revealing the mechanism of mitochondrial transfer between these two cell types and providing a new strategy for vascular regeneration [130]. Liu *et al*. elucidated that Dmp1-expressing astrocytes mediate mitochondrial transfer to endothelial cells via nanotubular protrusions, thereby preserving blood–brain barrier integrity. These findings suggest that maintaining mitochondrial homeostasis through mitochondrial transplantation may be a potential therapeutic approach in the nervous system [131]. Jiang *et al*. reported that iPSC-derived MSCs effectively delivered functional mitochondria into retinal ganglion cells, which had a protective effect on retinal ganglion cells due to mitochondrial dysfunction and improved retinal function [132]. Research has indicated that in stroke-induced oxidative neuronal injury, the transfer of mitochondria from MSCs to neurons can improve the metabolism of neuronal cells [133]. Astrocytes facilitate the release of mitochondria into neurons, thereby promoting cell viability and recovery following stroke [134]. Emerging studies demonstrate that astrocytic LRP1 suppression impairs intercellular mitochondrial transfer to compromised neurons, consequently aggravating cerebral ischemia–reperfusion pathology. Clinical investigations further establish elevated cerebrospinal fluid lactate concentrations in stroke patients versus healthy controls, demonstrating an inverse association with astrocytic mitochondrial content within the cerebrospinal fluid (CSF). These findings suggest that LRP1 exerts a protective effect on cerebral ischemia–reperfusion injury through mitochondria-mediated astrocyte–neuron interactions [135]. Current evidence suggests that intercellular mitochondrial trafficking is orchestrated through specific signaling nodes and effector proteins, with therapeutic potential achievable via targeted modulation of key effectors to regulate transfer magnitude, thereby enabling therapeutic outcome optimization. Nevertheless, the impact of MSC mitochondrial transfer on peripheral nerves remains to be substantiated. Yao *et al*. elucidated that MSCs orchestrate precision mitochondrial transfer to neuronal populations, thereby augmenting post-SCI neurological restoration through ferroptosis inhibition [136]. Similarly, the therapeutic application of PNI relies on the verification of the mechanism whereby the mitochondrial transfer of MSCs inhibits iron-induced death in injured neurons. Some evidence is available from studies of optic nerve diseases that the mitochondrial transfer of MSCs is a viable approach. Optic nerve cells—including retinal nerve cells—are susceptible to oxidative stress, which frequently results in the progressive deterioration of the optic nerve and loss of vision. Once optic nerve ganglion cells are damaged, they are unable to regenerate. Consequently, exogenous mitochondrial transfer methods are of significant clinical importance for the repair of optic nerve damage. Figure 3 briefly presents the pathways and mechanisms by which mitochondrial transfer and transplantation promote peripheral nerve regeneration.

#### Mitochondrial transplantation

**Figure 4:**
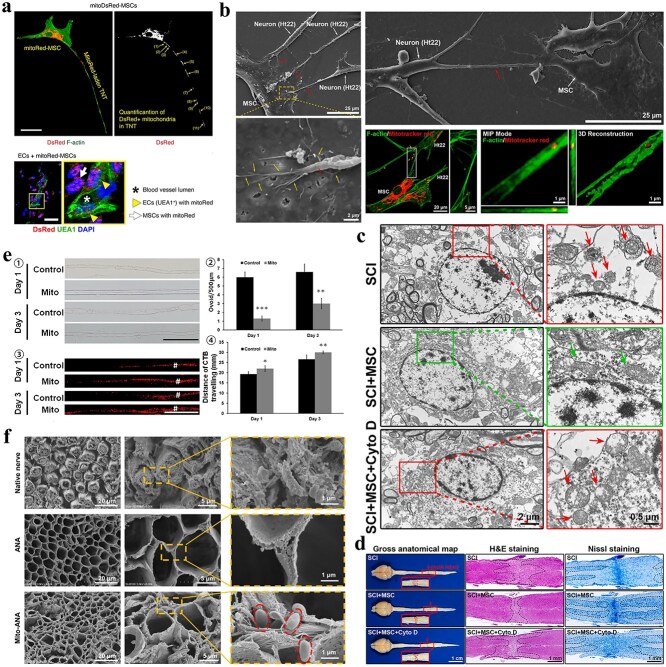
Intercellular mitochondrial dynamics and exogenous mitochondrial engraftment within peripheral neural regenerative paradigms. (**a**) MSCs orchestrate mitochondrial shuttle via TNTs-mediated intercellular transport (upper panel); scale bar, 10 μm; MSCs-derived mitochondria undergo intercellular transfer to endothelial cells (lower panel). DsRed^+^ mitochondria are labeled with white arrowheads (red fluorescence). UEA1^+^ ECs-harboring DsRed^+^ mitochondria are denoted by yellow arrowheads. Blood vessel lumens are marked with asterisks. Scale bars, 50 μm. (Reprinted with permission from ref. [130], © 2024 Springer Nature). (**b**) SEM images and representative confocal images of MSCs interacting with multiple neurons through short TNTs. MSC-derived mitochondria were fluorescently stained with MitoTracker Red. (**c**) TEM ultrastructural analysis of spinal cord neuronal mitochondria at 24 h post-SCI: Comparative profiles across SCI-only, MSC-transplanted, and MSC + CytoD intervention cohorts. Crimson/green chevrons demarcate pathological/intact mitochondrial ultrastructures, respectively. Cyto D is the potent actin polymerization inhibitor cytochalasin D (a blocker of TNT formation). (**d**) Representative gross anatomical maps and images of H&E and Nissl staining showing the lesion core and neuronal region 42 days after spinal cord injury in the SCI group, SCI + MSC group, and SCI + MSC + Cyto D group. (b–d: Reprinted with permission from ref. [136], © 2023 Elsevier B.V.) (**e**) Assessment of the early response of mitochondrial injection to nerve degeneration. ① and ② Representative images of ovoid changes and quantitative analysis of the number of ovoid nerves after an injection of 195 μg of mitochondria at 1 and 3 days after nerve injury. Scale bars, 100 μm. ③ Distribution of cholera toxin subunit B (CTB) and quantitative analysis of the distance traveled by CTB in injured nerves based on an examination of nerve integrity via retrograde CTB labeling. Scale bars, 25 μm (Reprinted with permission from ref. [137], © 2017 Crown copyright). (**f**) MSC-sourced mitochondria bioengineer ECM-based grafts to potentiate nerve defect regeneration. SEM images of native nerves, ANAs, and Mito-ANAs; the red dashed circle indicates mitochondria (Reprinted with permission from ref. [140], © 2023 Wiley-VCH GmbH)

After nerve injury, mitochondrial dysfunction occurs in neurons. Healthy mitochondria can be transported into damaged neurons by exogenous implantation strategies to help restore the energy balance, rescue mitochondrial dysfunction, and support cell survival. Kuo *et al*. showed that mitochondrial interventions were protective for injured nerves in both *in vitro* and *in vivo* experiments and that their effects may be related to attenuating cytoskeletal loss and reducing oxidative stress [137]. In optic neurotrauma models, mitochondrial transplantation ameliorated oxidative phosphorylation and restored bioelectrical activity within 24 h post-administration [138]. Sheu *et al*. demonstrated that localized mitochondrial administration in a rodent sciatic nerve crush model enhances neuromuscular histoarchitectural restoration, with concomitant improvement in locomotor functionality [139]. In peripheral nerve tissue engineering, mitochondria synergized with neural grafts demonstrate enhanced regenerative efficacy. hUCMSC-sourced mitochondria significantly boost SC proliferative, migratory, and respiratory capacities during coculture. Multi-omics analyses reveal that TCA cycle augmentation and metabolic reprogramming drive microenvironmental remodeling, achieving optimized bioenergetic output [140]. Zhang *et al*. demonstrated that mitochondrial administration upregulated ATF3 and associated gene networks in dorsal root ganglion neurons, significantly enhancing axonal regenerative capacity in murine sciatic nerve injury (SNI) models [141]. The ATF gene is highly expressed in injured and regenerating dorsal root ganglia [141], orchestrating the initiation of neural repair cascades while transcriptionally regulating recombinase-activated gene networks. Quantitative analysis of regenerating nerve fibers at Day 4 post-injury revealed statistically significant intergroup disparities between mitochondrial intervention and control cohorts; however, such differential outcomes were not sustained at later timepoints (Days 8/12). The authors posit that this difference may be associated with the timeframe in which mitochondrial integrity is maintained following a mitochondrial injection. Ongoing investigations must establish mitochondrial therapy’s therapeutic window optimization and develop sustained therapeutic efficacy protocols. Nerve regeneration extends beyond axonal regrowth, encompassing Schwann cell functionality, remyelination dynamics, and regenerative niche microenvironmental modulation. Therefore, we believe that the role of mitochondrial injection should be multifaceted and should focus not only on regenerative nerve fibers. However, the extent and scope of research in related areas still require further investigation. Emerging research reveals that adipocyte-derived leptin enables Schwann cells to orchestrate regenerative nerve catabolism—specifically modulating myelin autophagy and mitochondrial bioenergetics—to meet elevated metabolic requisites of neural repair [127]. Targeted modulation of Schwann cell bioenergetics emerges as a viable therapeutic strategy to enhance neural repair efficacy. Figure 4 delineates mitochondrial transfer dynamics and translational applications of mitochondrial grafting in neural injury management.

#### Immunomodulation of mitochondria

PNI pathogenesis induces an inflammatory microenvironment that partially compromises neural repair mechanisms. Mitochondria play pivotal roles in the inflammatory response, regulating oxidative stress and apoptosis. Additionally, they serve as a source of energy for immune cells through oxidative phosphorylation [142]. Immune cells can ingest and deliver pathogens to mitochondria by cytophagy, thereby facilitating the killing of pathogens localized during injury through the respiratory chain and ROS production. Mitochondria initiate the apoptotic pathway by releasing proapoptotic factors, such as cytochromes, with the objective of removing excess immune cells and controlling the number and function of immune cells [143].

In the context of injury, damaged mitochondria can release mtDNA, proteins, and other substances into the cytosol. These molecules function as damage-associated molecular patterns (DAMPs), eliciting innate immune activation through pattern recognition receptor (PRR) signaling. The release of mitochondrial DNA and reactive oxygen species can stimulate an immune response within the host cell, which, in turn, causes inflammation. Mitochondria orchestrate phagocytic and apoptotic mechanisms to mitigate DAMP generation while preserving organellar homeostasis and functionality [144]. Mitochondria represent the primary site of intracellular oxidative stress, and oxidative stress represents a crucial mechanism underlying inflammatory responses. During an inflammatory response, mitochondria release significant quantities of ROS. The level of oxidative stress within mitochondria can be regulated, which influences the extent of the inflammatory response. Mitochondria-derived cytokines such as TNF-α/IL-1β translocate to the cytosol, orchestrating inflammatory cascades via immunomodulatory signaling. Mitochondria engage endoplasmic reticulum networks to modulate Ca^2+^ dynamics and lysosomal interfaces to fine-tune inflammatory magnitude [145].

Mitochondrial dysfunction triggers cytoplasmic/extracellular compartmentalization of organellar components and byproducts, inducing inflammatory cascades. The cGAS-STING1 axis operates as the core regulatory hub for mitochondria-driven inflammation while concurrently serving as a central contributor to age-related chronic inflammation and physiological deterioration. Apoptotic and autophagic pathways constitute essential regulatory mechanisms in mitigating mitochondrial inflammatory pathogenesis [146]. Cytosolic translocation of mtDNA triggers NF-κB signaling pathway activation, upregulating proinflammatory mediator expression [147]. Additionally, mtDNA can directly activate NLRP3 inflammatory vesicles, which, in turn, activate the caspase-1 signaling pathway, leading to increased cytokine expression. Mitochondria can activate the cellular innate immune response by releasing mtDNA into the cytoplasm [148].

Mitochondrial autophagy represents an intracellular protective mechanism for the removal of damaged or abnormally functioning mitochondria. This process maintains mitochondrial quality and function and, in tissues, prevents excessive inflammatory responses, which can be exacerbated when mitochondrial autophagy is impaired [149, 150]. Shao *et al*. demonstrated that diacetyl sulfone alleviates chronic NP in murine sciatic nerve crush models by suppressing mitophagy-driven NLRP3 inflammasome activation in microglia. In addition, di(vanillin)sulfone restored the mitochondrial membrane potential by scavenging intracellular ROS [151]. Li *et al*. demonstrated that melatonin upregulates Parkin expression, sustains autophagic flux, suppresses neuronal apoptosis, and stimulates remyelination [152, 153]. Melatonin demonstrates significant therapeutic efficacy in murine peripheral nerve repair. Cytoplasmic fenugreek triggers mitochondrial DNA-enriched vesicle release, activating innate immunity. LKB1—a serine/threonine kinase—activates the AMPK pathway to modulate energy metabolism and mitochondrial dynamics, thereby regulating immunoinflammatory responses [154]. Furthermore, cellular physiological processes—including mitochondrial biogenesis, division, and fusion—provide the foundation for mitochondrial function.

While mitochondria are recognized as significant regulators of inflammation, additional research is needed to fully elucidate the underlying mechanisms involved. Although a substantial body of evidence linking inflammatory responses driven by mitochondrial dysfunction to disease exists, a clear mechanistic understanding is lacking [155].

### Mesenchymal stem cell secretome

Mesenchymal stem cell-conditioned medium (MSC-CM)—comprising the MSC secretome—is enriched with bioactive molecules (e.g. cytokines and growth factors) released by MSCs. Notably, MSC-CM has emerged as a pivotal cell-free therapeutic modality in neuroregeneration, circumventing tumorigenic risks, immunological rejection, and ethical constraints inherent to MSC transplantation. Proteomic profiling of diverse MSC lineages identified secretomes abundant in neurotrophic factors—VEGF, IGF, PDGF, and HGF—and anti-inflammatory cytokines [156]. Lv *et al*. did not identify clear fixed characteristics of the composition of secretomes, which vary considerably from one secretome to another, depending on factors such as the tissue source and culture conditions [156].

In peripheral nerve injury, the application of MSC-CM may act through neurotrophic factors to reduce cell death in neurons and satellite cells [157]. Analogously, BMSC-conditioned medium potentiates post-injury sciatic nerve myelin-axon regeneration via upregulating myelination-associated and neurotrophic factors while suppressing apoptotic pathways [158]. Yuan *et al*. suggested that MSC-CM may act through BDNF to exert neuroprotective and immunomodulatory effects on retinal ganglion cells without potential adverse effects [159]. In the context of chronic pain, Amodeo *et al*. suggested that the ADSC-derived secretome may favorably modulate neuroinflammation in the central and peripheral nervous systems and contribute to osteoarthritis-induced pain relief [160]. With respect to NP, Liu *et al*. investigated the function of hSHED-CM in a mouse sciatic nerve ligation model of nociceptive tactile hypersensitivity. Their findings indicated that hSHED-CM markedly suppressed the hypersensitivity response by recruiting M2-type macrophages to the injury site and in the vicinity of the ipsilateral dorsal root ganglion. Furthermore, M2-type macrophages recruited by hSHED-CM were shown to suppress the expression of Schwann cell injury receptors and proinflammatory mediators [161]. In light of these findings, the authors’ proposed secretome study provides a novel approach and insights into potential avenues for NP treatment. These findings suggest that BMSC-CM modulates anti-inflammatory and proinflammatory factors and exerts a potent and long-lasting nociceptive effect on NP. A comparative study was conducted by Yuriy Petrenko *et al*. using a combination of bone marrow MSCs. The secretome was compared, with a focus on the neuroregenerative potential of all three cell types: BMSCs, ADMSCs, and UCMSCs. The authors discovered that, among the three secretory groups, the BMSC secretory group exhibited the most robust immunomodulatory capacity and the lowest neurotrophic factor levels. However, the secretomes of all three cell types displayed a notable neurotrophic capacity to stimulate axonal growth in dorsal root ganglion cells and to mitigate oxidative stress-induced apoptosis in neural stem/progenitor cells [162]. Theoretically, the secretomes of different types of stem cells should contain different cytokines, microRNAs, and proteins. Therefore, proteomics, genomics, and bioinformatics can be used to characterize the relevant secretomes. We believe that the repair effects of the three types of stem cells should be evaluated and compared from the aspect of the final effect on neural repair in animal models. In the field of tissue engineering, the triad comprises seed cells, scaffold material, and cytokines. The secretome contains a plethora of cytokines that can be employed as part of a tissue engineering approach to facilitate nerve repair. Raoofi loaded BMSC-CM onto 3D polycaprolactone scaffolds for the treatment of mouse sciatic nerve dissection injury. The results of their study suggested that BMSC-CM acted to reduce the death of neurons and satellite cells through neurotrophic factors [157].

The secretome of DMSCs provides superior neuroprotection. DMSCs exhibit neurotrophic superiority over BMSCs and ADSCs through trophic factor enrichment. DMSCs orchestrate Schwann cell mitogenesis, chemotaxis, and lineage commitment via cytokines (IGF-1/BDNF), while PDGF/TGF-β signaling drives concurrent neurovascular regeneration. Collectively, DMSC-CM establishes a neuroprotective niche supporting axogenesis, myelin reformation, metabolic homeostasis, and antiapoptotic effects [163]. Although the secretome is effective in neuroprotection and regeneration, reducing inflammation, and improving function in experimental animal studies, researchers have found that the preparation of secretomes and the heterogeneity of different secretomes affect the translation of clinical research applications. Currently, no reports on the application of secretomes in clinical trials are available, and further research is needed to develop secretome-related therapies.

The secretome encompasses all cellularly secreted proteins into the extracellular milieu, comprising paracrine factors, microbubbles, and exosomes. Exosomes constitute a key constituent of this secretory repertoire. The role of exosomes is as described above, whereas the secretome, as a mixture, exerts a broader neuroregenerative effect.

### Organoids

Organoids are tissue analogs with a specific three-dimensional structure that are formed using adult stem cells or pluripotent stem cells in three-dimensional culture *in vitro*. While organoids lack anatomical equivalence to native human organs, they recapitulate organotypic architecture/functionality with high *in vivo* fidelity and sustain stable long-term maintenance [164]. Organoids represent the gold standard 3D systems for neurodevelopmental studies, disease pathogenesis modeling, and pharmacological delivery *in vitro*. These systems exist as two distinct classes: tissue-originated organoids and pluripotent stem cell-originated organoids [165]. In this review, we briefly summarize the few studies related to MSC organoids and peripheral nervous system diseases.

Van Lent *et al*. prepared an organoid culture that mimics peripheral nerves and contains Schwann cells. This self-organizing organoid model with iPSCs can focus on specific parts of the many complex features of the disease and be developed as an *in vitro* model to study neural demyelination to better facilitate *in vitro* studies of this class of diseases [166]. One of the major challenges in organoid development is the formation of functional blood vessels that can provide nutrients to organoid cells. A lack of the vasculature can lead to organoid developmental immaturity, nuclear necrosis, and premature differentiation. Although translational issues in organoid research are still in their infancy and have not progressed significantly, their importance for experimental applications in biomedicine is undeniable. Organoid research has been partially groundbreaking in many neurodevelopmental, neuropsychiatric, and spinal cord injury disorders, but models for studying these disorders in the peripheral nervous system are still relatively scarce.

The manipulation of the ECM and gene regulation are highly important in the construction of organoids. Stem cell-derived organoids display the characteristics of the organ of origin, including cell types, functions, and tissue spatial structures. Furthermore, microfluidic systems, endothelial cell coverage modules, and vascular endothelial growth factor delivery systems have been shown to be effective in promoting the formation of blood vessels, thereby facilitating the transport of oxygen or nutrients to the interior of organoids. The study of organoids has evolved significantly over time, progressing from planar cell culture to 3D structured tissue culture and beyond. ECM research has been a key area of development, as has the creation of vascularization techniques and spatiotemporal regulation of signaling pathways [167, 168]. In recent years, organoids have been developed with customized construction methods, and genome engineering techniques to modulate iPSCs through gene editing can precisely regulate target genes, thus facilitating the development of *in vitro* models of disease and biomedical translation. In the locomotor system, motor neuron (MN) axons and muscle fiber endplates are correlated; together, they innervate the function of skeletal muscle and influence its development. The neuromuscular junction (NMJ) represents the point of contact between an MN and an endplate of a muscle fiber. It facilitates the transmission of neuroelectrical and chemical signals through synapses, neurotransmitters, and ion channels [169]. NMJ disorders frequently result in motor dysfunction, with a paucity of effective pharmacological treatments and animal models being unable to fully elucidate human pathophysiology. Consequently, robust *in vitro* models are clearly needed [170]. Among these models, stem cell-derived organoids are of particular importance. NMJ-like organs are formed through the stem cell-induced self-organization of MNs and skeletal muscle cells.

**Figure 5:**
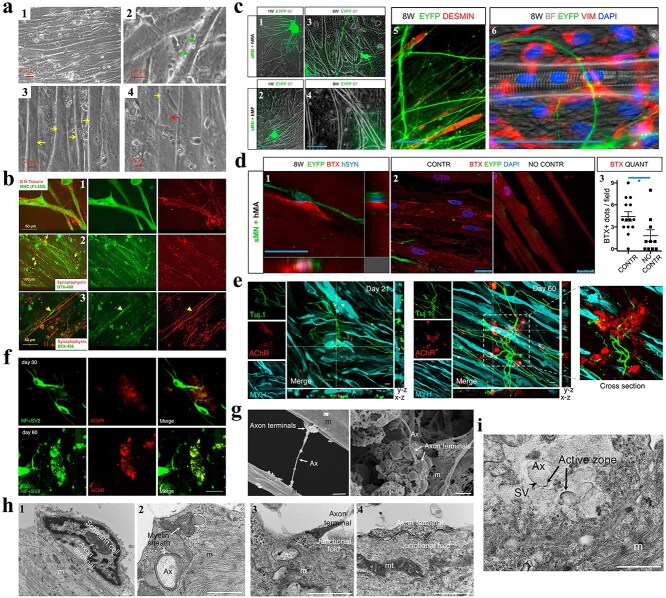
NMJ formation between human fetal spinal stem cell-derived motoneurons and skeletal muscle stem cell-generated myofibers. (**a**) 1: Cocultured myotubes and neurons maintained viable cytoarchitecture post-plating. 2: Neuronal-myotube synaptogenesis was demonstrated in coculture (white arrows). 3: Cross-striated myotubes (yellow arrow) confirmed mature differentiation in coculture. 4: MN-phenotyped neuron extending axonotrophic projections (red arrow) toward cross-striated myotubes (yellow arrow). (**b**) Synaptogenesis evidenced by immunocytochemical profiling. 1: Immunophenotyping of coculture constituents (Day 19): Dual MHC/B-III tubulin labeling revealed axonal arborization with perimyotube terminal ensheathment. 2 and 3: Synaptic interface mapping (Day 15): Synaptophysin+/BTX-488+ colocalization identified presumptive NMJs (arrows/arrowheads). (a and b: Reprinted with permission from ref. [171], © 2011 Elsevier Ltd). (**c**) Characterization of neuromuscular cocultures. 1 and 2: Cocultures of spinal hESC-derived MNs with adult (hMA)- and fetal (hMF)-derived myofibers at 1 W post-initiation; EYFP and bright field channels shown. 3 and 4: Cocultures of spinal hESC-derived MNs with hMA- and hMF-derived myofibers at 6–8 W. 5: Dense network of EYFP^+^ axons with desmin^+^ muscle fibers. 6: Multinucleated, striated myofibers closely associated with EYFP^+^ neuronal processes in contractile regions; scale bars, 100 μm. (**d**) NMJ formation in organoids. 1: High-magnification confocal imaging shows acetylcholine receptor clusters (BTXs) colocalized with EYFP+ neuronal processes and synaptophysin. 2 and 3: AChR clustering compared between contractile (CONTR, left) vs. non-contractile (NO CONTR, right) regions. The quantification of BTX^+^ dots revealed a significant increase in contracting/innervated regions. Scale bars, 25 μm. *P <0.05. (c and d: Reprinted with permission from ref. [172], © 2016 Elsevier Inc.) (**e**) Formation of human NMJs (hNMJs) from iPSCs in a single well *in vitro*, and the morphologies of pre- and postsynaptic features at Days 21 and 60 with *z*-axis views. Tuj1-labeled neurons in the presynaptic phase. AChR is labeled with a-BTX-647. MYН, skeletal muscle marker. Scale bars, 10 μm. (**f**) Morphology of AChR clustering and an NMJ at Days 30 and 60. NF, neurofilament. Scale bars, 10 μm. (**g**) Fine structures of hNMJs. SEM images demonstrating swollen axon terminals interfacing with a myotube. (**h**) 1 and 2: TEM imaging demonstrates synaptic boutons ensheathed by terminal Schwann cells and myelin. 3 and 4: enlarged axon terminals within myotubes; junctional folds appear as electron-dense zones. (**i**) TEM details presynaptic vesicles (arrowheads), active zones (arrows), and postsynaptic mitochondria enrichment. Scale bars, 1 μm (g–i). (e–i: Reprinted with permission from ref. [173], © 2019 American Society for Clinical Investigation)

Guo *et al*. demonstrated that hSCSC lines and hSkMPCs synergistically generate MN-skeletal muscle units, which after 7-day coculture exhibited physiological membrane excitability and contractile functionality. Additionally, well-defined and differentiated NMJs were observed [171]. Currently, scholars tend to utilize hESCs and iPSCs to model NMJs in two-dimensional cultures. Steinbeck *et al*. revealed that mature myotubes derived from hiPSCs were capable of inducing myoblast maturation and accumulating many acetylcholine receptors (AChRs) after 3 weeks of coculture with MNs. They showed that hiPSCs are capable of forming NMJ-like connections and can be readily detected. Nevertheless, further investigation is required to ascertain the functionality and morphology of this product [172]. Emerging research demonstrates that hiPSCs directly differentiate into mature myotubes via myogenic factor MYOD1 activation, bypassing conventional multicellular coculture requirements. The cells were subsequently transferred to a culture medium conducive to neuronal differentiation and maturation. The formation of structures such as synaptic nuclei, endplates, and AChRs was observed, indicating the successful construction of NMJs, which was subsequently verified [173]. In the context of organoids of NMJs, 3D culture provides a more accurate representation of the *in vivo* growth environment. Coculturing human myoprogenitors with hiPSC-differentiated MNs generated functional NMJs, validated through calcium flux imaging and electrophysiological assays [174]. Similarly, Martin *et al*. isolated SD rat-derived primary MNs and myocytes, coculturing them within 3D bioengineered fibronectin hydrogels to establish NMJ synaptogenesis. A fascicular arrangement of myotubes was observed via confocal microscopy, with axons extending from the cytosol of the ganglion to the myotubes [175]. Mazaleyrat *et al*. codifferentiated hiPSCs into skeletal muscle cells and MNs to induce the production of mature muscle fibers [176]. 3D organoid platforms orchestrate MN-skeletal myocyte-Schwann cell crosstalk, thereby driving NMJ developmental maturation. Figure 5 demonstrates functional NMJ synaptogenesis between human iPSC-derived MNs and engineered skeletal muscle constructs.

### Extracellular matrix

For large segmental nerve defects, autologous nerve grafts remain the clinical gold standard. However, limitations persist with this method, prompting the development of tissue-engineered nerve grafts as emerging viable alternatives. From a bionics perspective, nerve grafts based on the ECM have unparalleled advantages over other synthetic grafts.

**Figure 6:**
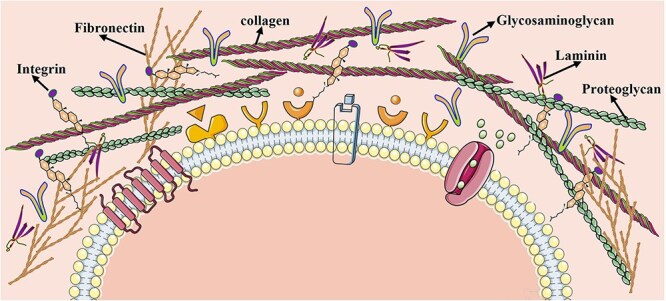
Schematic of key ECM constituents: fibronectin, collagen, integrin, glycosaminoglycan, proteoglycans, and laminin, functioning as a tissue engineering scaffold. The figure was drawn by the author

The ECM—a cell-secreted network extracellularly deposited in tissues—comprises two molecular classes: fibrous proteins (collagen, elastin) and glycoproteins (fibronectin, proteoglycans, and laminin) [177]. Figure 6 illustrates the ECM components functioning as biomaterial scaffolds in tissue engineering. The ECM enables stem cell/cytokine delivery while creating regenerative spaces for injured nerves. It provides critical biophysical functions: mechanical support, structural connectivity, and compression resistance. Additionally, the ECM orchestrates cellular processes including shape modulation, survival/apoptosis balance, and growth regulation while directing cellular migration and differentiation [178, 179]. A reasonable conclusion is that the ECM is highly bioactive and can be used as an ideal material for regenerative medicine. Furthermore, its use as a scaffold for grafts represents a highly competitive option. Emerging research demonstrates that TREM2-driven ECM remodeling induces mitochondrial fragmentation and dysfunction in human fibroblasts. The TGF-β signaling pathway mediates TREM2-induced mitochondrial remodeling, specifically enhancing fission processes. This ECM–mitochondrial interaction mechanism may be involved in immune regulation in a tissue-specific manner [180]. Wang *et al*. demonstrated BMSC-ECM-modified nerve grafts (NGs) functioned as neurotrophic factors, modulating immune responses to optimize the nerve injury microenvironment and enhance repair. The design can be personalized in accordance with the intended application, with the additional benefit of low immunogenicity, by preparing the ECM from the patient's own stem cells [23]. The use of decellularized nerve allografts in sciatic nerve defects in rats and sheep has been reported to promote nerve growth [181].

**Figure 7:**
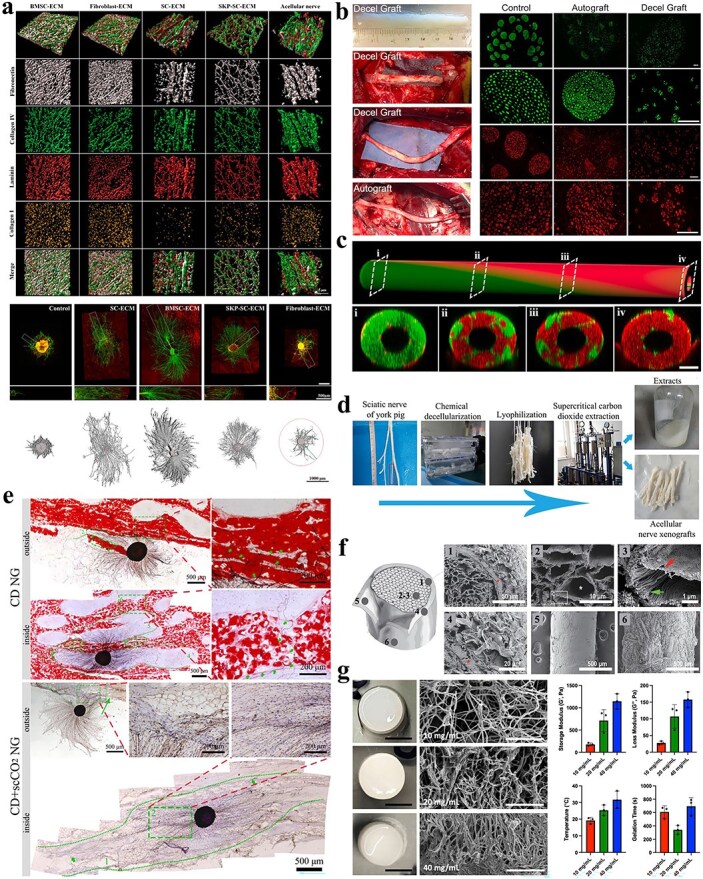
Cell-derived and multiple forms of tissue-derived ECM promote peripheral nerve regeneration. (**a**) 3D immunostaining images of fibronectin (white), laminin (red), collagen I (gold), and collagen IV (green) in various ECM sources with Imaris software analysis (upper). Scale bars: 5 μm. Lower panel: DRG axonal outgrowths (NF-200, green) on SC-, BMSC-, SKP-SC-, fibroblast-derived ECM (laminin, red) *vs* PLL control, with a schematic demonstrating NF-positive neurite branch quantification methodology (lower panel). (Reprinted with permission from ref. [23], © 2021 Elsevier Ltd). (**b**) Repair of a 7-cm common peroneal nerve defect in sheep with allogeneic decellularized nerve grafts and immunofluorescence staining of mid-portion cross-sections of the grafts at 9 months postsurgery, together with autologous nerve grafts as controls (NF-200, green; S100, red); the scale bar at the top is 200 μm; all others are 100 μm. (Reprinted with permission from ref. [181], © 2022 MDPI.) (**c**) A novel microtube that contains a gradient decellularized porcine sciatic nerve ECM hydrogel (pDScNM-gel) from microfluidics, and the cross-sectional CLSM images of the microtube revealed that it not only maintained a homogeneous core–shell structure but also showed gradient compositions in different regions. Scale bars, 200 μm. (Reprinted with permission from ref. [183], © 2022 Elsevier B.V.) (**d**) Flowchart for the preparation of porcine-derived acellular nerve xenografts via supercritical extraction technology. (**e**) NF200 immunohistochemistry and Oil Red O staining of the axonal growth state of dorsal root ganglia growing on the membrane of purely chemically decellularized nerve grafts and supercritical extracted nerve grafts. (d–e: Reprinted with permission from ref. [182], © 2022 Elsevier B.V.) (**f**) SEM characterization of decellularized nerve matrix scaffold (DNM-S) ultrastructure: (1–4) Cross-sectional views showing DNM-S (1), endoneurium (2,3; green arrow), and perineurium–epineurium regions (4; red asterisk). Myelin/axonal components removed (white asterisk), while endoneurial tubes (red arrow) and perineurium (red asterisk) preserved. (5 and 6) Contrasting surface morphology: Medial perineurium smooth; lateral epineurium textured. (Reprinted with permission from ref. [185], © 2023 Wolters Kluwer Medknow Publications.) (**g**) Characterization of decellularized nerve-specific hydrogels. Macroscopically, hydrogels formed across all concentrations with enhanced fluid retention capacity at higher protein levels. Scale bars: 0.5 cm. SEM analysis of 10, 20, and 40 mg/ml gels revealed concentration-dependent increases in fiber density. Scale bars: 2.5 μm. Rheological profiles across concentrations demonstrate storage/loss moduli; gelation temperature/time parameters. (Reprinted with permission from ref. [184], © 2021 Nature Publishing Group)

With respect to tissue-derived ECM, research on decellularized porcine neurogenic ECM is more prevalent. This approach is employed to create hydrogels that are used to fill the gaps in nerves and enhance nerve regeneration. Previous research by our group has shown that the treatment of porcine peripheral nerves using supercritical extraction composite technology to maximize the removal of DNA and fat from NGs can facilitate enhanced cell adhesion and proliferation, thereby rendering it a decellularization method with considerable clinical applicability [182]. Jin *et al*. developed gradient-decellularized porcine sciatic nerve ECM hydrogels with microchannel-incorporated fluids to repair nerve defects [183]. Decellularized porcine nerve-derived hydrogel filler was applied to treat large segmental nerve defects. The matrix preserved multiple nerve-specific components and growth factors, yielding therapeutic advantages for nerve repair [184]. Decellularized nerve matrix scaffolds inhibit painful neuroma formation post-sciatic nerve transection in rat models of NP. This property has been evidenced by a notable reduction in the level of pain experienced by the animals, accompanied by the provision of a regenerative microenvironment conducive to the recovery of injured nerves [185]. Thus, the ECM's role in peripheral nerve injury primarily stems from regulating the regenerative microenvironment [186], promoting Schwann cell proliferation/migration and axonal growth. Decellularized grafts of neural tissue—when combined with BMSCs or ADSCs—have been transplanted into the peripheral nerve defect area, resulting in a more significant immunomodulatory effect when ANAs are used in conjunction with BMSCs. Furthermore, the combination of ANAs and ADSCs has been shown to have a more significant pro-myelination and angiogenic effect, and the two together have been shown to activate different mechanisms [20]. Zheng *et al*. demonstrated that neural guidance conduits (NGCs) comprising 0.25% pDNM gel-modified oriented PLLA nanofibers facilitate axon extension, myelin sheath formation, and neurological recovery [187]. The ability of cell-derived and tissue-derived ECM to promote peripheral nerve regeneration is shown in Figure 7.

The ECM serves as neural conduits/graft scaffolds to modulate regenerative microenvironments during neural repair [188]. Tissue-engineered nerve grafts based on the ECM show great potential as a substitute for autologous nerve transplantation. However, ECM-related tissue engineering products have problems with implant immunogenicity and application standardization, especially for tissue-derived ECM. Cell-derived ECM products may be promising raw materials for future tissue engineering products [189]. Future research directions focus on: (i) investigating ECM heterogeneity across cell sources and its mechanisms driving cellular behaviors/tissue regeneration, (ii) optimizing ECM components through genome editing to enhance bioactivity/mechanical properties by adjusting composition/structure/modifications, and (iii) engineering multicellular coculture systems incorporating Schwann/vascular endothelial/neural stem cells within ECM scaffolds mimicking *in vivo* microenvironments, enabling dynamic biomechanical/biochemical simulation via multicell-derived ECM.

### miRNA

As previously stated, microRNAs (miRs) may be responsible for modulating the relevant regenerative signaling pathways in peripheral nerve regeneration therapy using MSCs. miRs are endogenous noncoding RNAs (~22 nt) mediating posttranscriptional regulation in organisms. Multiple miRs critically regulate post-injury nerve regeneration. Transcriptomics identified upregulated miR-21/132/29a/29b post-sciatic injury, modulating stress responses and epidermal growth factor receptor (EGFR) signaling. Upstream regulators (miRs, including let-7, miR-21 and miR-223) correlate with PNI-induced transcriptional dysregulation, demonstrating essential roles in peripheral nerve repair [190].

The involvement of miRs in myelin formation and synaptic plasticity has been documented. Ma *et al*. demonstrated MSC-mediated suppression of Ras/Akt/GSK-3β signaling via exosomal miR-132-3p enrichment activating RASA1, rescuing neuronal/synaptic dysfunction [191]. Wu *et al*. reported that, following recurrent laryngeal nerve injury in rats, scaffolds grafted with filled MSCs exerted their reparative effects through miR-21-mediated activation of Notch signaling.

miRs critically regulate immune function and neuronal differentiation. Wang *et al*. demonstrated BMSC-mediated peripheral nerve repair through miR-449a targeting, NF-κB modulation, and inflammation suppression [192]. miRs such as miR-27/125/145 mediate MSC-driven neuronal differentiation [193]. Gong *et al*. reported that the delivery of let-7 miRs from MSCs to endothelial cells via extracellular vesicles enhances angiogenesis [106]. This effect enhances PNI revascularization post-MSC transplantation. Concurrently, Chen *et al*. demonstrated that let-7 functions as a key modulator of nerve regeneration, with multimodal actions on Schwann cells, macrophages, and fibroblasts [194]. Notably, miRs can also regulate the functions of stem cells in many ways. Emerging evidence demonstrates that MSC-derived miRs modulate aging phenotypes while enhancing neural repair/regeneration through functional stem cell regulation [195, 196]. miRs expressed in iPSCs can promote self-renewal and maintain pluripotency [197].

The involvement of microRNAs (miRs) in myelin formation and nerve regeneration has been documented, and miRs can exert their regulatory functions by acting on Schwann cells. miR-21 overexpression downregulates PTEN and activates PI3K, driving Schwann cell phenotypic reprogramming to enhance axonal regeneration and post-injury functional recovery [198]. Following nerve injury, miR-21 exerts a proproliferative and axonal regenerative effect on Schwann cells through TGF-β1, TIMP3, and EphA4 [199]. Among the miRs involved in Schwann cell formation, the MAPK, AKT, and EGR2 pathways may play significant roles [200]. Cong *et al*. demonstrated hypoxia-preconditioned BMSC-neural crest cell-derived EVs enhance sensory axon regeneration via miR-21-5p cargo [201]. Hypoxia-preconditioned BMSC exosomes enhance facial nerve repair via the circRNA_Nkd2/miR-214-3p/Med19 axis by stimulating Schwann cell proliferation/migration and boosting growth factor secretion capacity [91]. Zhao *et al*. demonstrated that BMSC-derived exosomes facilitate peripheral nerve regeneration and are associated with the miRs-mediated regulation of regeneration-related genes [22]. Following PNI, neuronal cytosolic damage or apoptosis occurs at the damaged site, and maintaining the activity of damaged neurons to preserve their inherent regenerative capacity is essential. Suppression of miR-192-5p upregulates X-associated apoptosis inhibitors, thereby attenuating neuronal apoptosis and enhancing regeneration in injured sciatic nerves [202].

Notably, the lifespan of miRs is relatively short, which may limit their continuous effects during treatment. However, through an appropriate carrier or drug delivery system, the duration of their effects can be prolonged to a certain extent, and their effectiveness in PNR can be improved [203]. Moreover, elucidating miRs *in vivo* dynamics requires further investigation to decipher their biological impacts. Advancing the precision therapy of miRs demands engineered delivery systems (nanoparticles/liposomes) to enhance target-specific transport precision; exploring combined therapeutic strategies; combining miRs therapy with other therapeutic methods such as gene editing and drug therapy to exert a synergistic effect; and developing personalized medical plans, such as formulating personalized miRs treatment plans based on the individual genetic information, disease characteristics, and miRs expression profiles of patients, which is the future direction.

### Combination effects of derivatives

Whether derivatives synergistically promote nerve regeneration when used in combination is a question worth pondering. MSCs exert different effects under different conditions, and manual intervention, such as the simultaneous extraction of mitochondria and exosomes from MSCs, may be required if combination therapy is needed.

#### Construction and regulation of the cellular microenvironment

ECM and secretome collaboratively establish a pro-regenerative microenvironment. Secretome-derived growth factors/cytokines modulate ECM biosynthesis/secretion, while ECM reciprocally adsorbs/enriches the secretome’s bioactive molecules, creating localized high-concentration niches [204]. Secretome-derived TGF-β upregulates MSC collagen secretion and enhances ECM deposition, creating a matrix-enriched niche that bolsters neural cell survival/proliferation/migration.

#### Neural cell metabolism and functional support

Mitochondria-secretome interplay sustains neuronal energy/functional support. Secretome-derived IGF-1 enhances mitochondrial internalization within neuronal populations, while mitochondria provide ATP to neuronal cells to maintain their energy requirements during regeneration [205]. Secretome components modulate neuronal mitochondrial functions (e.g. oxidative phosphorylation) to enhance cellular functional recovery [128].

#### Gene expression regulation and cell differentiation guidance

miRs and exosomes cooperate to regulate gene expression in neuronal cells at the posttranscriptional level [97]. Exosomes act as carriers of miRs and can transport MSC-derived miRs into neuronal cells. miRs operate within neurons to direct NSC fate toward neuronal/glial lineages [206]. Exosomal miR-124 drives NSC neuronal differentiation, while other cargo components sustain differentiated neurons, synergizing nerve regeneration [207].

Multicomponent synergy (MSC-exosomes/secretome/mitochondria/ECM/miRs derivatives) potentiates neural regeneration via establishing regenerative niches, sustaining neuronal homeostasis, and epigenetic modulation. While preclinical evidence demonstrates efficacy, clinical translation requires validation of these combinatorial mechanisms.

## Conclusions

Stem cell transplantation emerges as a promising therapeutic strategy, with clinical trials demonstrating early-phase benefits in patient cohorts. Current studies indicate MSC derivatives (exosomes/mitochondria/ECM) elicit comparable therapeutic efficacy to parental MSCs. However, the current theories and techniques regarding cell therapy application and clinical translation have not yet fully matured, which has become an obstacle to the clinical translation of MSCs and their derivative therapies. Thus, MSC and derivative developmental trajectories center on these key aspects: (i) In-depth research must be conducted and standardized protocols optimized. Innovative extraction and purification techniques are needed. In the future, a pressing need is to develop more efficient and precise extraction and purification methods for MSCs and their derivatives. (ii) Research on cellular and molecular mechanisms is needed. Further exploration of the interactions between MSCs and their derivatives and the immune and vascular systems during neural regeneration is necessary. (iii) Exploring and implementing combined therapeutic strategies is a third approach. Synergistic therapeutic regimens for MSCs and other cells (e.g. neural stem cells and Schwann cells) or biologically active factors, along with rational combinations of MSCs with other cells with the potential to repair nerves (such as neural stem cells and Schwann cells) or biologically active factors, can achieve synergistic effects. (iv) These treatments should be integrated organically with traditional therapies. The modes of combined application of MSCs and their derivative treatments with surgical, physical, and pharmacological therapies and other traditional treatment modalities should be explored. Simultaneously, formulating clinical application guidelines is necessary to clarify issues such as cell sources, preparation processes, administration routes and dosages, and treatment timing.
